# Quantification of Cell-Free DNA in Normal and Complicated Pregnancies: Overcoming Biological and Technical Issues

**DOI:** 10.1371/journal.pone.0101500

**Published:** 2014-07-02

**Authors:** Irina Manokhina, Tanjot K. Singh, Maria S. Peñaherrera, Wendy P. Robinson

**Affiliations:** 1 Department of Medical Genetics, University of British Columbia, Vancouver, British Columbia, Canada; 2 Child & Family Research Institute, Vancouver, British Columbia, Canada; Warwick University, United Kingdom

## Abstract

The characterization of cell-free DNA (cfDNA) originating from placental trophoblast in maternal plasma provides a powerful tool for non-invasive diagnosis of fetal and obstetrical complications. Due to its placental origin, the specific epigenetic features of this DNA (commonly known as cell-free fetal DNA) can be utilized in creating universal ‘fetal’ markers in maternal plasma, thus overcoming the limitations of gender- or rhesus-specific ones. The goal of this study was to compare the performance of relevant approaches and assays evaluating the amount of cfDNA in maternal plasma throughout gestation (7.2–39.5 weeks). Two fetal- or placental- specific duplex assays (*RPP30/SRY* and *RASSF1A/β-Actin*) were applied using two technologies, real-time quantitative PCR (qPCR) and droplet digital PCR (ddPCR). Both methods revealed similar performance parameters within the studied dynamic range. Data obtained using qPCR and ddPCR for these assays were positively correlated (total cfDNA (*RPP30)*: R = 0.57, *p* = 0.001/placental cfDNA (*SRY)*: R = 0.85, *p*<0.0001; placental cfDNA (*RASSF1A)*: R = 0.75, *p*<0.0001). There was a significant correlation in *SRY* and *RASSF1A* results measured with qPCR (R = 0.68, *p* = 0.013) and ddPCR (R = 0.56, *p* = 0.039). Different approaches also gave comparable results with regard to the correlation of the placental cfDNA concentration with gestational age and pathological outcome. We conclude that ddPCR is a practical approach, adaptable to existing qPCR assays and well suited for analysis of cell-free DNA in plasma. However, it may need further optimization to surpass the performance of qPCR.

## Introduction

The quantification and characterization of cell-free DNA (cfDNA) has opened up new possibilities for prenatal diagnosis and screening [1], as well as for monitoring diseases such as cancer [2]. During pregnancy, cfDNA derived from the apoptotic and necrotic placental trophoblast cells can be found in the maternal circulation in addition to DNA released from maternal cells [3]. The ability to measure DNA from the ‘fetus’ (specifically, placenta [4]) in maternal blood has introduced new possibilities for non-invasive prenatal diagnosis (NIPD), allowing for the diagnosis of aneuploidies [5], monogenic diseases [6], and early determination of fetal sex or RhD status [7], [8]. In addition, quantitative assessment of placental DNA in maternal plasma has been associated with clinical and biological parameters, offering the potential for the prediction of pregnancies at risk of preeclampsia, intra-uterine growth restriction (IUGR) or preterm labour [9]–[11].

The concentrations of placental cfDNA and maternal cfDNA in maternal plasma vary between individuals. The amount of maternally derived cfDNA can vary depending on maternal health factors, such as obesity or diabetes [12]. Placenta-derived cfDNA level is affected by gestational age and the health of the placenta; for example, aneuploidy or preeclampsia can alter trophoblast growth and death dynamics [13]. In conditions such as preeclampsia, both maternal and placental cfDNA increase, hence the absolute quantification of cfDNA, but not the ratio, may be predictive of disease onset. However, there can be technical confounders in the quantification of cfDNA, including storage conditions or processing delay before plasma separation [14]–[16], DNA extraction method [17]–[20], amplicon size and target gene choice [21]–[23]. Moreover, there are no generally accepted units of measure for cfDNA quantification; in the literature data are presented in genome equivalents per mL of plasma (GE/mL) [21], [24]–[27] or its logged equivalent [27], median C_T_ [28], [29], percent value to total cfDNA [30], [31] or multiple of median (MoM) [11]. This inconsistency complicates the data analysis, comparability and reproducibility of the tests.

Quantification of placental DNA can be performed in male-bearing pregnancies using Y chromosome-specific sequences such as *SRY* [9], [10], [32]. To extend this approach to all pregnancies (i.e. both male and female fetus bearing pregnancies), it has been proposed to take advantage of locus-specific DNA methylation differences found between placental trophoblast cells and maternal blood cells [33], [34]. This approach requires the additional step of methylation-sensitive restriction enzyme digestion, which prevents the amplification of hypomethylated targets of maternal origin. A third approach is to take advantage of genomic sequence differences between the fetus and mother. Massive parallel sequencing has yielded promising results in this regard given the nature of fetal DNA where the majority of the fragments of interest are longer than the average read length (25–100 base pairs) [5], [35]. However, the cost is high for diagnostic testing and thus not yet applicable for screening purposes. Additionally, while this approach can provide data on the ‘fetal’ fraction (placental/total DNA), parallel sequencing is not applicable for absolute quantification which is a more relevant parameter for prediction of placental dysfunction or preterm birth.

Two major approaches currently exist for absolute quantification of placental cfDNA: real-time quantitative PCR (qPCR) and digital PCR. While qPCR requires calibration of the magnitude of the signal ( ΔRn) across the reaction using a standard curve, digital PCR technology is based on direct counting the number of positive signals in a partitioned reaction [36]. The potential advantage of digital droplet PCR (ddPCR) approach [37] is its high reproducibility at the dynamic range relevant for cfDNA assessment and its higher sensitivity, allowing detection of a single target molecule per well, along with easy lab setup.

The goal of this study was to compare the performance of real-time quantitative and droplet digital PCR for the quantification of total and placental cfDNA in maternal plasma across gestation (7.2–39.5 weeks) using two placental-specific assays (*RPP30/SRY* and *RASSF1A/β-Actin*).

## Materials and Methods

### Subjects

The samples used in this study were ascertained as part of a study on preeclampsia and IUGR. This study was approved by the University of British Columbia Clinical Research Ethics Board (H04-70488). Patients provided written consent; the consenting process has been approved by the Board. Whole blood was collected from 38 pregnant women between 7.2 and 39.5 weeks of pregnancy by venipuncture using Vacutainer tubes. The tubes were stored at room temperature for 1–30 h before processing. Whole blood was centrifuged at 4°C for 10 min at 3 000 *g*. The supernatant (plasma) was transferred to a new tube and centrifuged for 10 min at 16 000 *g* at 4°C. The supernatant plasma was transferred to a new tube and stored at −80°C until DNA extraction. Initial plasma volume, time before separation and pregnancy outcome (normal/pathological, fetal sex) was recorded for 33 samples which were included in the study group (Table S1).

### Cell-free DNA extraction

Two DNA extraction protocols were used in this study. In the first set of 26 samples obtained, 1.8–2.4 mL of plasma DNA were extracted using the DNeasy Blood & Tissue Kit (Qiagen, Valencia, CA), *nonnucleated blood* protocol and eluted in 400 µL of elution buffer. We then extracted DNA from 20 plasma samples (2.5–5 mL) using the QIAamp Circulating Nucleic Acid Kit (Qiagen, Valencia, CA), which is designed to enrich for cell free DNA. Extraction was done according to the manufacturer’s recommendations and the DNA was eluted in 30 µL of the supplied elution buffer. In order to compare the yields between the two extraction protocols, 8 samples were extracted using both methods. The difference was assessed using a paired sample *t*-test. A high correlation between placental cfDNA levels was obtained for these two extraction approaches (C_corr_ = 0.85, *p* = 0.015, qPCR *RASSF1A* data were evaluated). While the placental cfDNA yield from the QIAmp kit on average was higher than the yield from the DNAeasy kit, this difference was not significant based on this small number of samples (*p* = 0.18). The total cfDNA levels (qPCR *RPP30* assay) obtained using the QIAmp kit were approximately 10% higher than those with DNeasy kit (*p* = 0.04), with a positive correlation between yields from both extractions though this was not statistically significant (C_corr_ = 0.65, *p* = 0.08), Tables S2, S3. While the differences may be significant with a larger sample size, for the purpose of the present study we included samples extracted with both kits (using the QIAamp extraction data for the study group).

#### PCR Assays

Two duplexed PCR assays were used in this study: 1) *RPP30/SRY* and 2) *RASSF1A/β-Actin* (Applied Biosystems, Foster City, CA; for primer and probe sequences and references see Table S4). In the duplex *RPP30/SRY* assay, the amplification of *RPP30* (located at 10q23) evaluates the total amount of cfDNA in the extracted plasma sample while the *SRY* amplification quantifies male (non-maternal) DNA. In the duplex *RASSF1A*/*β*-Actin assay, each sample was first treated with methylation-sensitive restriction enzymes *Hha*I (60 U), *Bst*UI (30 U) and *Hpa*II (30 U) (New England Biolabs, Whitby, ON), which have in total 8 restriction targets per each amplicon. The mixtures were incubated at 37°C for 2 h, 60°C for 2 h, and then 65°C for 20 min as previously described [37]. Since the *RASSF1A* promoter is hypermethylated in placental trophoblast and unmethylated in maternal blood [33], only placental DNA *RASSF1A* will not be cut by the above restriction enzymes, whereas maternal *RASSF1A* will be fully digested. *β-Actin* was used as a digestion control of the same amplicon size and with the same number of restriction sites as the *RASSF1A* region analyzed, and should not show specific amplification, since it is unmethylated in fetal, placental and maternal DNA.

*Serial dilutions*: A 7-point standard curve was generated using a DNA sample obtained from a normal male term placenta (chorionic villi) and diluted to a concentration comparable to the expected amounts of placental and maternal cfDNA [21], [25], [37]. DNA concentration for this sample was measured using a Nano Drop ND-1000 Spectrophotometer (Fisher Scientific Company, Ottawa, ON). These dilution series were amplified using both duplex assays, to evaluate their performance parameters. For the *RASSF1A*/*β-Actin* assay, each sample prepared for the standard curve was treated with methylation-sensitive restriction enzymes, as described before.

*Plasma DNA load:* In order to reach the placental DNA target concentration of at least 2 placental targets per well, based on the data of previous studies [21], [25], [37], approximately 1000 GE per well were loaded for all assays. The experiments were set up in quadruplicate for qPCR and in duplicate for ddPCR. For the samples coded 170, 173, 178, 181, 193, 196, 203, 213, 214, 216, 217, 220, 221, 229, 235, 239, 243, 254 the intended plasma load in the ddPCR experiment was reached by merging the data from two wells.

#### Approaches

*Quantitative real-time PCR.* Quantitative PCR was performed on an ABI 7500 system (Applied Biosystems, Foster City, CA) in a *Quantitation-Standard Curve* experiment. For each amplification reaction, the volume of purified total DNA calculated to at least 1000 GE (20 µL for Qiagen Blood & Tissue Kit extraction eluates in 50 µl total reaction volume and 0.5–1 µL for QiAmp Circulating Blood and Tissue Kit extraction eluates in 20 µl total volume) was added to a reaction mixture containing 1X *TaqMan Universal Master Mix II, no UNG* (Applied Biosystems, Foster City, CA), 600 mM of forward and reverse primer and 180 mM of probe. The thermocycler parameters were as follows: denaturation for 10 min at 95°C, followed by 15 sec at 95°C and 1 min at 60°C for 45 cycles. The 7-point standard curve, as well as a positive control (non-digested DNA for *RASSF1A*/*β-Actin*) and non-template controls (restricted whole blood for *RASSF1A*/*β-Actin*, plasma DNA from female-bearing pregnancy for *RPP30/SRY* and a reaction containing water in place of DNA for both) were included in all assays.

*Droplet digital PCR.* All ddPCR reactions were performed using the QX100 Droplet Digital system (Bio-Rad, Pleasanton, CA) in an *Absolute Quantification (ABS)* experiment, according to the manufacturer’s instructions. For each reaction, an equal volume of DNA (in *RPP30/SRY* assay) or product of restriction (*RASSF1A*/*β-Actin* assay) was added to a reaction mixture containing 1X ddPCR Mix for Probes (Bio-Rad, Pleasanton, CA), 900 mM of forward and reverse primer and 250 mM of probe in a total volume of 22 µL. Twenty µL of the reaction mixture were transferred into a cartridge and droplets were generated using a QX100 Droplet Generator. The droplets were transferred into a 96-well reaction plate (Eppendorf Canada, Mississauga, ON) and immediately sealed and loaded into a C1000™ Thermocycler (Bio-Rad, Pleasanton, CA). The PCR parameters were applied as suggested by the manufacturer: denaturation for 10 min at 95°C, followed by 30 sec at 95°C and 1 min at 60°C for 50 cycles, and final hold for 10 min at 95°C. Positive control (non-digested DNA for *RASSF1A*/*β-Actin*) and non-template controls (restricted whole blood for *RASSF1A*/*β-Actin*, plasma DNA from female-bearing pregnancy for *RPP30/SRY* and a reaction containing water in place of DNA for both) were included in all assays.

Data were obtained using QX100 Reader and analyzed with QuantaSoft 100 software (Bio-Rad). The mean number of droplets per well was 12711**±**1686 across all plates, this range does not include random wells with unexpectedly low droplet counts (6626**±**1769, 7.5%).

#### Data analysis

Data obtained in all experiments were translated into a GE/mL format using a conversion factor of 3.3 or 6.6 based on the weight of the human genome.

Statistical analysis was performed using SPSS Statistics for Windows, Version 19.0. (Armonk, NY: IBM Corp). Normality was assessed using the Shapiro-Wilk test (Table S5). Pearson’s correlation was used to determine correlation between positive qPCR and ddPCR values as well as between *SRY* and *RASSF1A* concentrations and biological parameters.

Alternatively, the Bland-Altman test [38] was used to evaluate a bias and agreement between the results for each comparable pair (i.e. qPCR(*RPP30*) *vs.* ddPCR(*RPP30*); qPCR(*SRY*) *vs.* ddPCR(*SRY*)*;* qPCR(*RASSF1A*) *vs.* ddPCR(*RASSF1A*); qPCR(*SRY) vs.* qPCR(*RASSF1A*) and ddPCR(*SRY*) *vs.* ddPCR(*RASSF1A*).

## Results

The overall goal of this study was to assess and compare the available approaches for routine quantification of placental cfDNA in maternal plasma.

### Question 1. Which method has better performance for placental cfDNA analysis?

#### Performance parameters for standard dilutions

We analyzed the performance parameters for two assays and two techniques using a 7-point standard curve. The standard dilutions were prepared to represent the range of expected DNA concentrations for cfDNA samples (2.3–9438 GE/well) and were run in quadruplicate. Both methods revealed linearity within the studied dynamic range (DR), although reported DR for ddPCR is more narrow than for real-time qPCR (5 vs. 9 log units) [37]. The regression coefficient used to test linearity of the standard curves was close to 1 for both assays and methods studied (qPCR R^2^ (*RPP30/SRY*) = 0.996/0.975, R^2^ (*RASSF1A*) = 0.981; ddPCR R^2^ (*RPP30/SRY*) = 0.998/0.998, R^2^(*RASSF1A*) = 0.952, to assess standard deviation ranges see Figure S1), indicating linearity and correlation between measured values. However, in the qPCR reaction, linearity was lost at the lowest dilution (2.3 GE per well) for the *SRY* assay, indicating that there is lower precision of measured values at this marginal end of the dynamic range. Fluorescence amplitude was reduced at the highest concentration (9438 GE per well) in the ddPCR *RPP30/SRY* assay (Figure S2), indicating lower precision, which is in agreement with developer’s instructions and can be overcome by additional restriction enzyme treatment [37]. At the lowest dilution (2.3 GE per well) specific amplification (qPCR) was detected in 4/4 (*RPP30),* 2/4 (*SRY*) and 0/4 *RASSF1A* wells and at least one positive droplet (ddPCR) was detected in 3/4 (*RPP30),* 2/4 (*SRY*) and 1/4 *RASSF1A* wells.

#### Evaluation of clinical samples

Although the developers report that ddPCR had a smaller DR than qPCR [37], in our experimental setup this method demonstrated successful quantification over the range of target concentrations used for both total and fractional quantification (mean **±** SD, log_10_(GE/mL): total cfDNA *(RPP30,* 3.09**±**0.34), placental cfDNA (*SRY,* 1.74**±**0.431) with no need of an additional restriction step.

**Figure 1:**
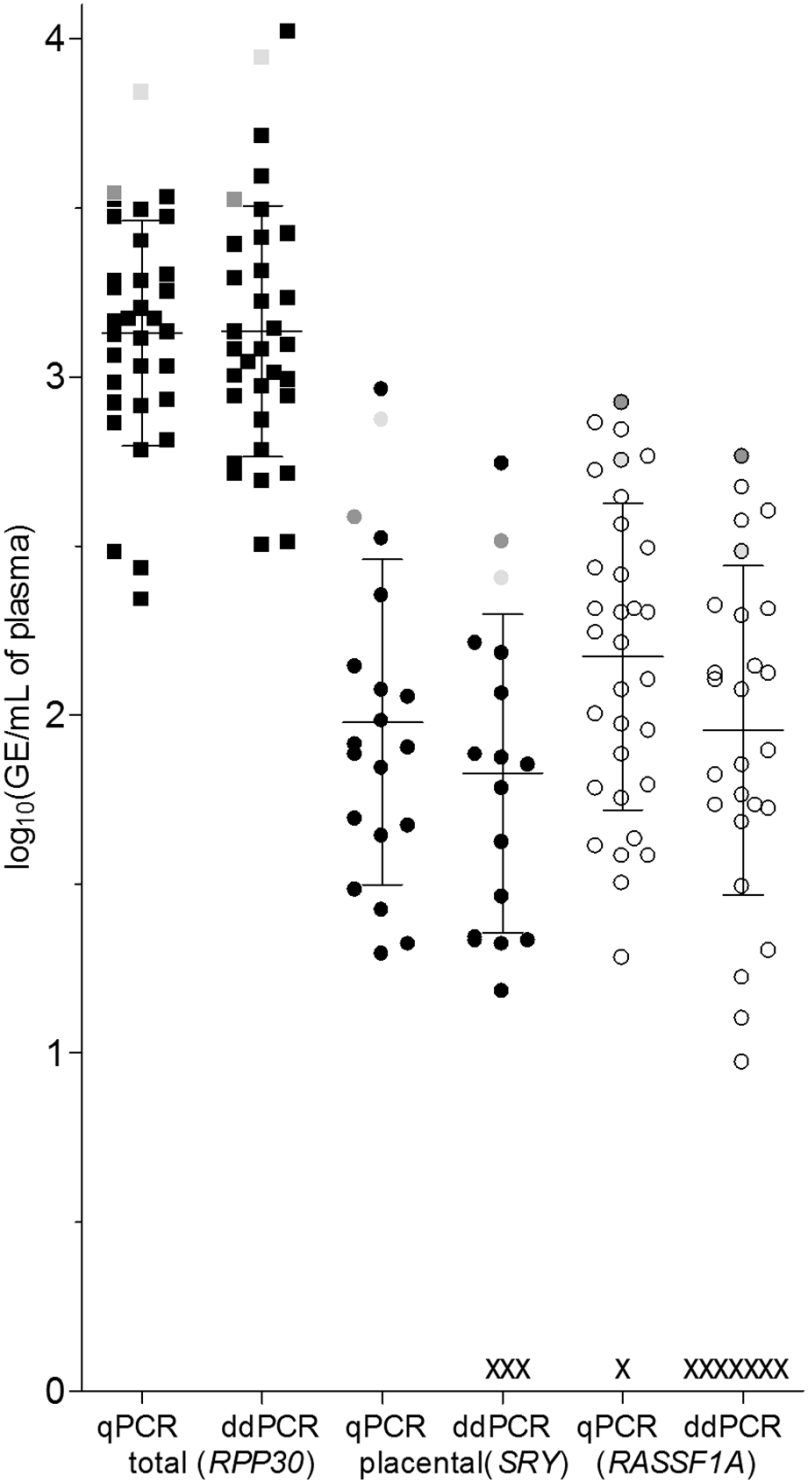
Quantification of total and fetal cfDNA with *RPP30/SRY* and *RASSF1A/β_Actin*-specific assays using qPCR and ddPCR. All values are presented in logged (GE/mL) and bars represent mean and standard deviation. Specific values: Light grey - Preeclampsia with HELLP syndrome at 29th week of gestation, dark grey - IUGR in twins. Each “x” represents a data point that has not been detected with the given approach.

The ddPCR assay revealed several false-negative results, which we attribute to a different experimental set-up compared to qPCR, such as fewer replicates or smaller maximum load volume per well, a limitation also highlighted by another group [39]. For the number of positive wells for the given samples in qPCR replicates see Table S6. Both PCR approaches produced comparable trends with regard to biological parameters such as gestational age and pathological outcome (Table S7, Figure S3). Samples obtained from women with IUGR, preeclampsia and/or in late-gestation (but not pregnancies complicated with NTD in fetus) revealed the highest placental cfDNA concentrations, which all exceeded one SD from the mean for that assay using both PCR methods (Figure 1). These results are in agreement with previously published data [9], [10], [21], [24], [27] but do not provide enough evidence to establish conclusions as only a few pathological samples were available for the study. We detected a positive association between gestational age and the concentration of placental fraction for both qPCR results (*SRY*, R = 0.49, *p* = 0.04, n = 18; *RASSF1A*, R = 0.45, *p* = 0.013, n = 30) and ddPCR *RASSF1A* data (R = 0.47, *p* = 0.022, n = 24), (Table S7). We also detected a significant association between placental and total cfDNA concentrations (Table 1, correlations marked with ‡). We did not find significant associations between total plasma DNA concentration and pre-processing delay of less than 30 hours (independent sample *t*-test for equality of means, group 1 (<10 hrs, n = 23) and 2(>20 hrs., n = 10); *p* = 0.075 for qPCR(*RPP30*), *p* = 0.99 for ddPCR(*RPP30*) data.

**Table 1:** Correlation (Pearson’s) between different DNA concentration values for *RPP30*, *SRY* and *RASSF1A* assays using qPCR and ddPCR techniques.

Assays	*RPP30,* ddPCR	*SRY,* qPCR	*SRY,* ddPCR	*RASSF1A,* qPCR	*RASSF1A,* ddPCR
*RPP30* q	**0.57 (** ***p*** ** = 10^−3^, n = 33)** *	0.42 (*p* = .06, n = 20)‡	0.36 (*p* = .15, n = 17)	**0.46 (** ***p*** ** = .008, n = 32)** ‡	**0.60 (** ***p*** ** = 10^−3^, n = 26)**
*RPP30* dd	-	**0.57 (** ***p*** ** = .009, n = 20)**	**0.73 (** ***p*** ** = 10^−3^, n = 17)** ‡	0.43 (*p* = .015, n = 32)	**0.64 (** ***p*** ** = 5×10^−4^, n = 26)** ‡
*SRY* q	0.57 (*p* = .009, n = 20)	-	**0.85 (** ***p*** ** = 2×10^−5^, n = 17)** *	**0.68 (** ***p*** ** = .013, n = 19)** †	**0.56 (** ***p*** ** = .03, n = 15)**
*SRY* dd	0.73 (*p* = 10^−3^, n = 17)	0.85 (*p* = 2×10^−5^, n = 17)	-	**0.69 (** ***p*** ** = .003, n = 16)**	**0.56 (** ***p*** ** = .039, n = 14)** †
*RASSF1A* q	0.43 (*p* = .015, n = 32)	0.68 (*p* = .013, n = 19)	0.69 (*p* = .003, n = 16)	-	**0.75 (** ***p*** ** = 10^−5^, n = 26)** *

Cells include Pearson’s R, *p*-values (statistically significant correlations with *p*<0.05 are in bold) and n (number) of samples.

*correlation between approaches (technical),

† correlation between assays,

‡ possible biological associations between total cfDNA and its ‘fetal’ fraction.

### Question 2. How comparable are the data obtained using qPCR and ddPCR?

While both assays appear reliable and reproducible, we wanted to know how the two quantification approaches correlated with each other. Data obtained using real time qPCR and ddPCR were positively correlated for total cfDNA (*RPP30)*: R = 0.57, *p* = 0.001/placental cfDNA (*SRY)*: R = 0.85, *p* = 2*10^−5^; and also for placental cfDNA (*RASSF1A)*: R = 0.75, *p* = 10^−5^, Table 1 (correlations marked with *) and Figure S4 (A).

**Figure 2:**
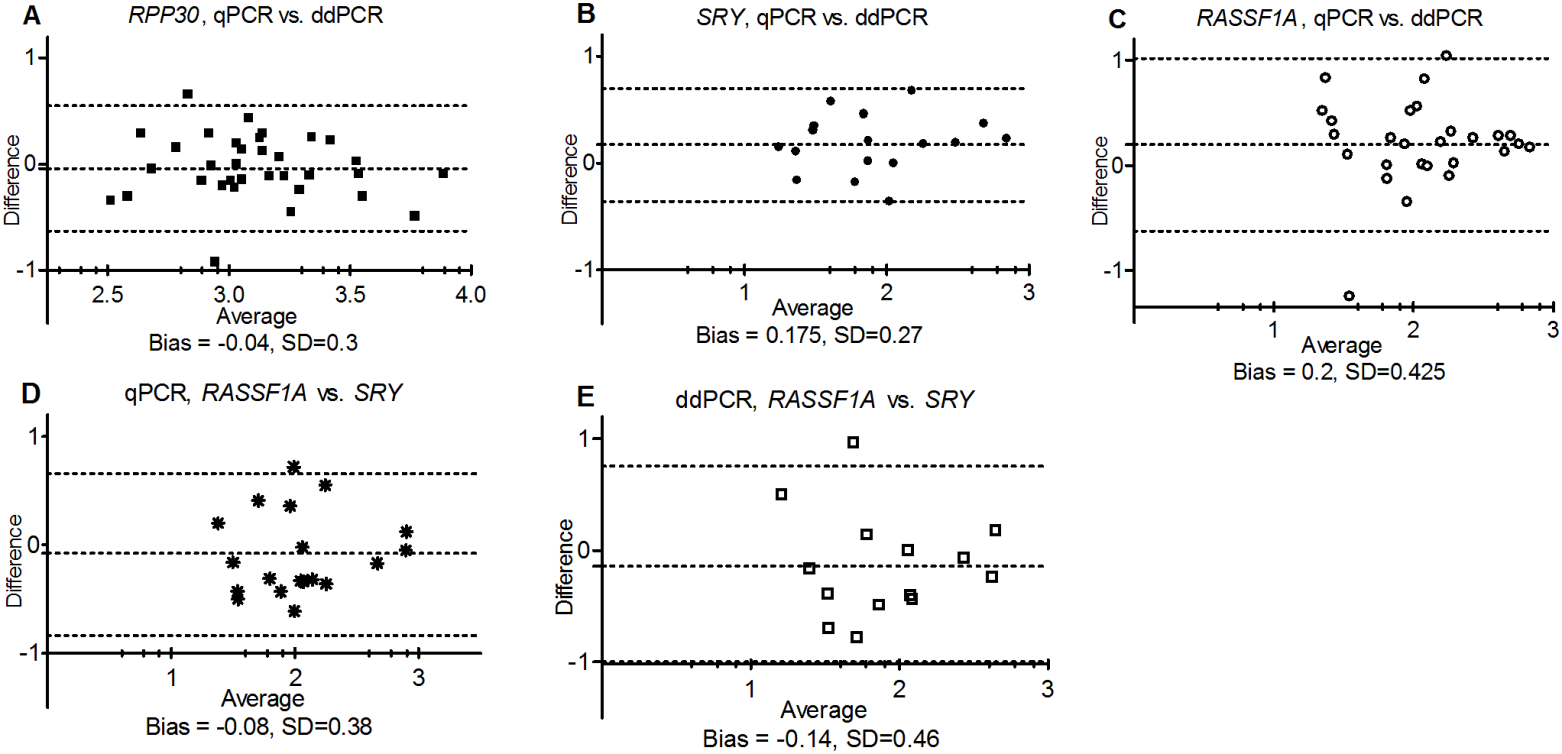
Difference (Bland–Altman) plots between two assays and two approaches. X-axis represents average value between two methods, Y-axis – difference between values, log_10_(GE/mL). Dotted lines indicate bias (mean difference) and 95% limits of agreement (2 SD) between the two given methods. A, B, C plots compare the results obtained with qPCR and ddPCR for *RPP30, SRY* and *RASSF1A* respectively; D, E compare the performance of *SRY* and *RASSF1* assays for qPCR and ddPCR.

Difference (Bland-Altman) plots were used to evaluate bias and the standard deviation between approaches (Figure 2 A, B, C). Placental cfDNA assays revealed a bias towards higher values obtained with qPCR. These higher values could be explained by the set-up of the absolute quantitation qPCR experiment, where the concentrations for the calibration curve were obtained by measuring the fluorescence absorbance of the DNA sample, whereas ddPCR amplifies only the intact targets of the highly fragmented template. *RPP30* and *SRY* reveal comparable SD values between the approaches which are smaller than that for the *RASSF1A* assay; this may be a consequence of using the additional digestion step in qPCR experiment.

### Question 3. How comparable are the values obtained using RASSF1A and SRY assays?

The *RASSF1A* assay represents a more universal approach given that it can detect cfDNA from both male and female fetuses; however, its utilization is dependent on a restriction enzyme digestion step, which represents a potential confounder in the case of incomplete digestion. *SRY*, as any Y chromosome-specific sequence, is an abundant fetal-specific marker that is not competitive with sequences in the maternal background; however, it is clearly not applicable for all pregnancies.

The quantification data was significantly correlated between *SRY* and *RASSF1A* assays for both qPCR (R  = 0.68, *p* = 0.013) and ddPCR (R = 0.56, *p* = 0.039), see Table 1, correlations marked with †, and Figure S4 (B); these data are comparable with the data obtained by another group [40]. We did not observe *RASSF1A* amplification in one sample (289, GA = 7.2 wks, processing delay 6 hours) where amplification was detected for *SRY*. This could be due to a number of factors including 1) a lower methylation level of placental trophoblast during the 1^st^ trimester, 2) DNA degradation during the incubation with methylation-sensitive restriction enzymes or 3) a lesser sensitivity of *RASSF1A* assay at the limiting dilutions. For difference plots, evaluating bias and the standard deviation between assays see Figure 2 (D, E). Both approaches revealed higher absolute values for *RASSF1A* assays, which cannot be explained biologically, and most likely is a consequence of quantification errors at limiting concentrations.

## Discussion

This study provides an assessment of the relevant approaches and assays for absolute fractional quantification of cfDNA in maternal plasma. The measurement of cfDNA in maternal plasma broadens the possibilities for prenatal diagnosis and pregnancy monitoring, but poses specific technical and biological challenges unique to cell-free nucleic acids in biological fluids.

Potential implementation of placental cfDNA analyses for diagnostic purposes generates a need for generally accepted units and standardized, accurate assays. The major challenge of the fractional quantification of fetal (placental) DNA in maternal plasma has been successfully overcome by the implementation of methylation-specific approaches [34]. In our study we detected a significant correlation between the outcomes of two assays (*RASSF1A* and *SRY*) using either real time or droplet digital PCR techniques.

A persistent challenge to the implementation of absolute quantification is that the detection of scarce targets may be influenced by both biological and technical factors. With the currently used approaches, placental DNA is accurately detectable in maternal plasma by the 6–11th week of gestation [7], [41], [42]. In one plasma sample obtained at 7.2 weeks gestation, we were able to amplify *SRY* but not *RASSF1A* with both PCR techniques. This is likely explained by lower trophoblast methylation during early pregnancy [33] and/or a lower sensitivity limit of the *RASSF1A* assay. The development of novel assays for which methylation is high in the placenta throughout gestation could improve results.

*SRY* and *RASSF1A* were moderately positively correlated using both PCR approaches. The *RASSF1A* promoter is a useful marker, though its use requires a constant restriction control (e.g. positive *β-Actin* amplification in 2.5% samples with qPCR and 18.5% with ddPCR possibly related to incomplete digestion was the reason for reassessment of a sample). Furthermore *RASSF1A* methylation could be affected by maternal oncological history [8], and more data is needed regarding potential variation in trophoblast methylation with pregnancy complications.

The placental cfDNA enrichment with pre-amplification may represent an additional confounding factor, but using the cell-free specific approach can potentially increase cfDNA yield for individual samples [18], [20], [43], [44].

The new PCR-era digital droplet PCR technology enables quantification of absolute DNA concentrations via digital measurements instead of calibration of an analog signal with a standard curve. It is proposed that partitioning of the genomic targets within the sample prevents reaction inhibition, allows for the detection of single targets, and also permits data analysis of a sample in a single well, avoiding additional dividing of the data in replicates [37]. Based on these factors we were expecting a higher performance of the ddPCR technology, but were not able to achieve this with our current reaction set-up. We experienced the following issues: first, we performed ddPCR analyses in duplicate in a merging format in order to: 1) equilibrate the plasma/product of restriction volume per sample or avoid false-negative calls due to limited load volume and 2) avoid low quality data obtained from wells with a total droplet count of <10000 (this accounted for 7.5% of wells). Second, single positive droplets of equal or exceeding amplitude to that of the target were occasionally detectable in non-template controls; this observation has been published before [39]. This observation, together with higher rate of positive *β-Actin* amplification may also suggest higher sensitivity of the digital droplet approach, nevertheless, we had a total of 9 false-negative results when quantifying placental cfDNA fraction with ddPCR (3 with *SRY* and 6 with *RASSF1A* assay). It is important to note, however, that for all but one sample in which the placental fraction was not detected with ddPCR, specific amplification was also missing in one or more of the qPCR replicates. It is possible that a different ddPCR experimental set-up, with an increased number of replicates analyzed (up to seven, [37]), would lead to better results in regards to sensitivity. In most cases this issue could be overcome with a higher plasma DNA load, however, assessing this was not the primary goal of our study. Additionally, the ddPCR data presented in this study were obtained using the first emerging technology with setup in the lab (Bio-Rad QX100 system) and thus cannot be extrapolated to alternative systems.

Despite these challenges, ddPCR was found to be a practical and efficient approach for the quantification of placental cfDNA in maternal plasma. There are readily available TaqMan assays for analyzing fetal-originating DNA that are suited for the analysis of cfDNA in plasma; though they may need additional optimization in order to surpass the performance parameters of the standard qPCR.

## Supporting Information

Figure S1 **qPCR (X axis, mean ± SD, logged) and ddPCR (Y axis, mean ± SD, logged) performance in seven 4x dilution series.** Four replicates are used for each data point and error bars represent the standard deviation.
(TIF)

Figure S2 **Visual representation of ddPCR results for serial dilutions.** The Y axis represents the fluorescent intensity and the X axis shows the number of events (positive events are above the threshold line). Each plot represents the data from one channel (Ch1: FAM (*SRY*), Ch2: VIC (*RPP30*)). B01 Non-template control (water), B02–B08 – Seven four-time dilutions within a range of 9438–2.3 GE/mL.
(TIF)

Figure S3 **Evaluation of placental cfDNA for individual samples over a range of gestational ages.** Specific values: *-Preeclampsia with HELLP Syndrome at 29th week of gestation, †-IUGR in twins, ‡-Neural tube defect.
(TIF)

Figure S4 **Correlation plots. A.** Logged concentrations of total (*RPP30*) and placental (*SRY*, *RASSF1A*) cfDNA obtained using qPCR and ddPCR. **B.** Logged concentrations of placental cell-free DNA measured with *SRY* and *RASSF1A* using different PCR techniques.
(TIF)

Table S1 **Case summary (patient samples with corresponding processing time, gestational age, total and fractional placental DNA concentrations measured with qPCR and ddPCR).**
(DOCX)

Table S2 **qPCR evaluation of 8 samples purified with both DNeasy Blood&Tissue Kit and QIAamp Circulating Nucleic Acid Kit.**
(DOCX)

Table S3 **Paired sample** ***t*** **-test for qPCR** ***RPP30*** **and** ***RASSF1*** **measurements for two DNA extraction approaches.**
(DOCX)

Table S4 **Primers and probes used in the study.**
(DOCX)

Table S5 **Normality test (Shapiro-Wilk).**
(DOCX)

Table S6 **Number of wells revealing specific amplification in qPCR assay in the samples negative for placental DNA according to ddPCR data.**
(DOCX)

Table S7 **Correlation of cfDNA levels with the gestational age at blood draw (GA).**
(DOCX)
