# Supplementary figures and images for: Quantification of Cell-Free DNA in Normal and Complicated Pregnancies: Overcoming Biological and Technical Issues
Source: PLoS One. 2014 Jul 2;9(7):e101500. doi: 10.1371/journal.pone.0101500 (PMC4079713; doi:10.1371/journal.pone.0101500)

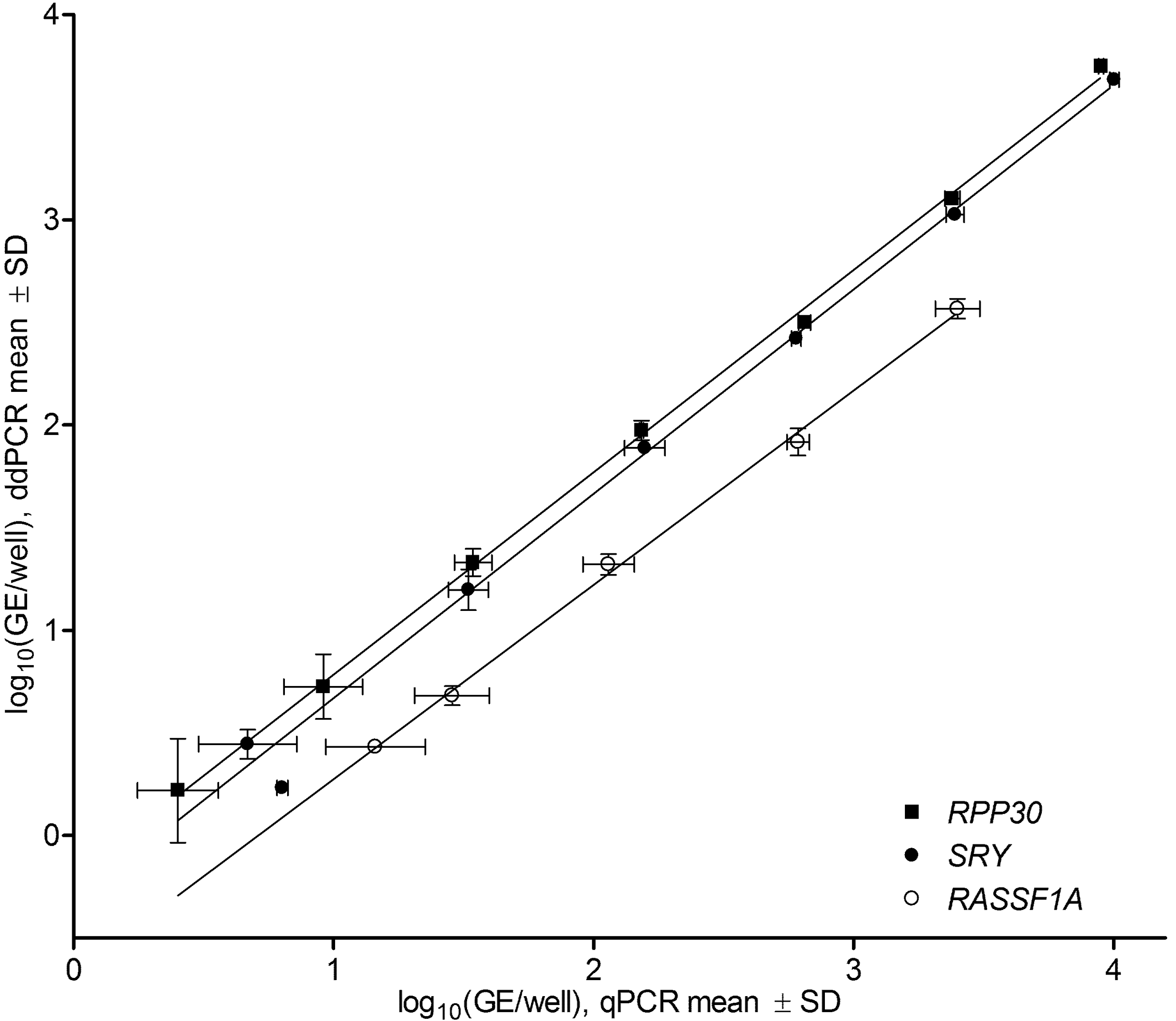

Supplement: Figure S1 — qPCR (X axis, mean ± SD, logged) and ddPCR (Y axis, mean ± SD, logged) performance in seven 4x dilution series. Four replicates are used for each data point and error bars represent the standard deviation. (TIF) [file pone.0101500.s001.tif]

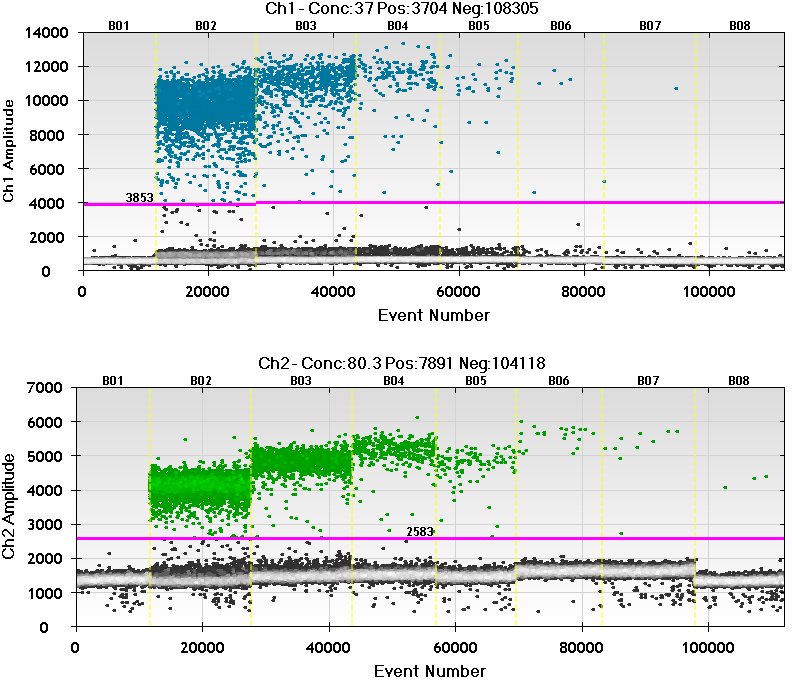

Supplement: Figure S2 — Visual representation of ddPCR results for serial dilutions. The Y axis represents the fluorescent intensity and the X axis shows the number of events (positive events are above the threshold line). Each plot represents the data from one channel (Ch1: FAM (SRY), Ch2: VIC (RPP30)). B01 Non-template control (water), B02–B08 – Seven four-time dilutions within a range of 9438–2.3 GE/mL. (TIF) [file pone.0101500.s002.tif]

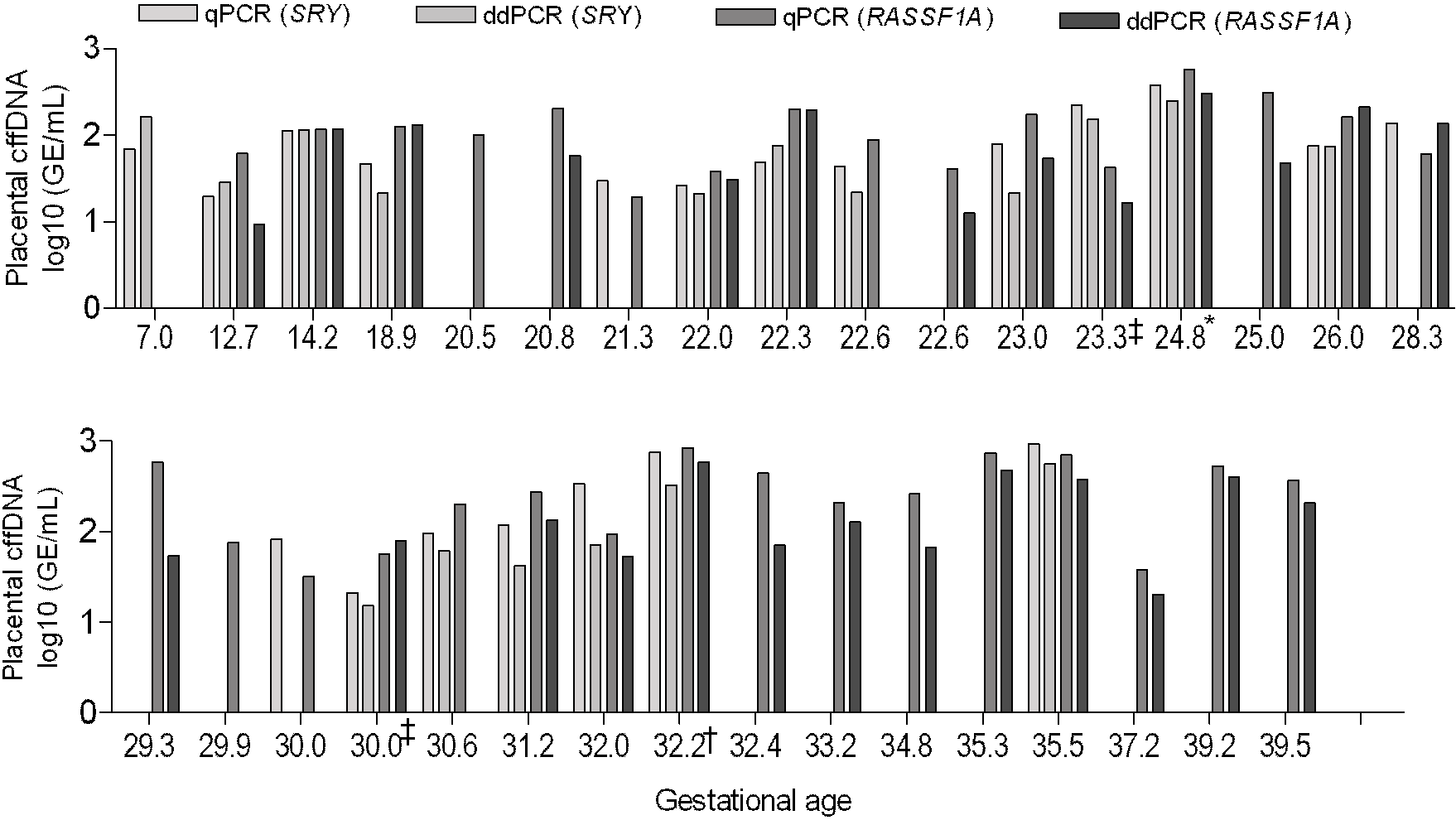

Supplement: Figure S3 — Evaluation of placental cfDNA for individual samples over a range of gestational ages. Specific values: *-Preeclampsia with HELLP Syndrome at 29th week of gestation, †-IUGR in twins, ‡-Neural tube defect. (TIF) [file pone.0101500.s003.tif]
