# Supplementary material for: Quantification of Cell-Free DNA in Normal and Complicated Pregnancies: Overcoming Biological and Technical Issues
Source: PLoS One. 2014 Jul 2;9(7):e101500. doi: 10.1371/journal.pone.0101500 (PMC4079713; doi:10.1371/journal.pone.0101500)
Supplement: Table S1 — Case summary (patient samples with corresponding processing time, gestational age, total and fractional placental DNA concentrations measured with qPCR and ddPCR). (DOCX) [file pone.0101500.s005.docx]

**Supplementary Table S1. Case summary (patient samples with corresponding processing time, gestational age, total and fractional fetal DNA concentrations measured with qPCR and ddPCR).** Data are presented in log_10_ (GE/mL). Samples with abnormal pregnancy outcome are marked with:

* - Preeclampsia with HELLP Syndrome at 29th week of gestation, † IUGR in twins, ‡ Neural tube defect. The samples purified with Qiagen Blood&Tissue Kit are in bold. n/a represents a value that has not been detected (and, subsequently, log transformed) with the given approach.

|  | Processing (hrs) | Gestational age (wks) | Total cfDNA | | Placental cfDNA | | | |
| --- | --- | --- | --- | --- | --- | --- | --- | --- |
|  |  |  | *(RPP30)* | | *(SRY)* | | *(RASSF1A)* | |
| Name |  |  | qPCR | ddPCR | qPCR | ddPCR | qPCR | ddPCR |
| **170** | 27.5 | 26 | 3.03 | 3.49 | 1.88 | 1.87 | 2.21 | 2.32 |
| **173*** | 4.5 | 24.8 | 3.84 | 3.94 | 2.58 | 2.4 | 2.75 | 2.48 |
| **178** | 1.7 | 21.3 | 3.17 | 3.29 | 1.48 | n/a | 1.28 | n/a |
| **181** | 23 | 14.2 | 3.28 | 3.39 | 2.05 | 2.06 | 2.07 | 2.07 |
| **193** | 4 | 25 | 2.91 | 3.14 | n/a | n/a | 2.49 | 1.68 |
| **196†** | 3.5 | 32.2 | 3.54 | 3.52 | 2.87 | 2.51 | 2.92 | 2.76 |
| **203** | 4.2 | 22 | 3.11 | 3.23 | 1.42 | 1.32 | 1.58 | 1.49 |
| **213** | 4 | 18.9 | 2.92 | 2.94 | 1.67 | 1.33 | 2.1 | 2.12 |
| **214** | 1 | 20.8 | 2.98 | 3.13 | n/a | n/a | 2.31 | 1.76 |
| **216** | 0.8 | 28.3 | 2.93 | 3.09 | 2.14 | n/a | 1.78 | 2.14 |
| **217** | 24 | 22.3 | 3.4 | 3.71 | 1.69 | 1.88 | 2.3 | 2.29 |
| **220** | 1.7 | 23 | 3.03 | 3.04 | 1.9 | 1.33 | 2.24 | 1.73 |
| **221** | 25 | 30.6 | 3.12 | 2.99 | 1.98 | 1.78 | 2.3 | n/a |
| **229** | 1 | 29.3 | 3.2 | 3.08 | n/a | n/a | 2.76 | 1.73 |
| **235** | 4.5 | 31.2 | 2.81 | 2.97 | 2.07 | 1.62 | 2.43 | 2.12 |
| **239** | 6 | 22.6 | 3.3 | 2.87 | 1.64 | 1.34 | 1.95 | n/a |
| **243** | 8 | 29.9 | 2.43 | 2.74 | n/a | n/a | 1.88 | n/a |
| **254** | 0.5 | 20.5 | 2.34 | 2.69 | n/a | n/a | 2 | n/a |
| 258 | 2.5 | 32 | 3.17 | 3.42 | 2.52 | 1.85 | 1.97 | 1.72 |
| 261 | 5 | 22.6 | 2.78 | 2.5 | n/a | n/a | 1.61 | 1.1 |
| 262 | 1.5 | 35.3 | 3.49 | 3.59 | n/a | n/a | 2.86 | 2.67 |
| 264 | 23 | 39.2 | 3.53 | 3.31 | n/a | n/a | 2.72 | 2.6 |
| 267 | 2 | 35.5 | 3.52 | 4.02 | 2.96 | 2.74 | 2.84 | 2.57 |
| 270 | 3 | 33.2 | 3.28 | 3 | n/a | n/a | 2.31 | 2.1 |
| 273 | 26 | 32.4 | 3.47 | 3.08 | n/a | n/a | 2.64 | 1.85 |
| 275 | 6 | 12.7 | 2.86 | 2.71 | 1.29 | 1.46 | 1.79 | 0.97 |
| 276 | 25.5 | 39.5 | 3.16 | 2.51 | n/a | n/a | 2.56 | 2.31 |
| 277 | 23.5 | 34.8 | 3.26 | 2.71 | n/a | n/a | 2.41 | 1.82 |
| 285 | 6 | 37.2 | 3.06 | 2.78 | n/a | n/a | 1.58 | 1.3 |
| 289 | 6 | 7 | 2.48 | 3.41 | 1.84 | 2.21 | n/a | n/a |
| 290 | 30 | 30 | 3.13 | 2.94 | 1.91 | n/a | 1.5 | n/a |
| 13‡ | 27 | 30 | 3.47 | 3.22 | 1.32 | 1.18 | 1.75 | 1.89 |
| 14‡ | 2 | 23.3 | 3.25 | 3.01 | 2.35 | 2.18 | 1.63 | 1.22 |
