# Supplementary material for: Quantification of Cell-Free DNA in Normal and Complicated Pregnancies: Overcoming Biological and Technical Issues
Source: PLoS One. 2014 Jul 2;9(7):e101500. doi: 10.1371/journal.pone.0101500 (PMC4079713; doi:10.1371/journal.pone.0101500)
Supplement: Table S2 — qPCR evaluation of 8 samples purified with both DNeasy Blood&Tissue Kit and QIAamp Circulating Nucleic Acid Kit. (DOCX) [file pone.0101500.s006.docx]

**Supplementary Table S2. qPCR evaluation of 8 samples purified with both DNeasy Blood&Tissue Kit and QIAamp Circulating Nucleic Acid Kit.** Data is presented in log_10_ (GE/mL). *RPP30* data evaluates the total amount of cfDNA in plasma and the *SRY* and *RASSF1A* data quantifies placental DNA.

|  | **Qiagen Blood and Tissue Kit** | | | **QIAamp Circulating Nucleic Acid Kit** | | |
| --- | --- | --- | --- | --- | --- | --- |
| **Sample** | ***RPP30*** | ***SRY*** | ***RASSF1A*** | ***RPP30*** | ***SRY*** | ***RASSF1A*** |
| 266 | 3.76 |  | n/a | 3.6 |  | n/a |
| 271 | 3.87 | 2.71 | 2.6 | 4.24 | 3.12 | 3.13 |
| 273 | 3.28 |  | 2.38 | 3.47 |  | 2.64 |
| 274 | 3.31 |  | 2.79 | 3.84 |  | 2.69 |
| 276 | 3.1 |  | 2.35 | 3.16 |  | 2.56 |
| 277 | 3.33 |  | 2.33 | 3.26 |  | 2.41 |
| N15 | 2.9 | 2.49 | 1.7 | 3.43 | 2.55 | 1.54 |
| N16 | 3.42 | 2.77 | 2.54 | 4.03 | 2.46 | 2.35 |
