# Supplementary material for: Quantification of Cell-Free DNA in Normal and Complicated Pregnancies: Overcoming Biological and Technical Issues
Source: PLoS One. 2014 Jul 2;9(7):e101500. doi: 10.1371/journal.pone.0101500 (PMC4079713; doi:10.1371/journal.pone.0101500)
Supplement: Table S3 — Paired sample t -test for qPCR RPP30 and RASSF1 measurements for two DNA extraction approaches. (DOCX) [file pone.0101500.s007.docx]

**Supplementary Table S3.** **Paired sample *t*-test for qPCR *RPP30* and *RASSF1A* measurements for two DNA extraction approaches.**

|  | ***RPP30*** | | ***RASSF1*** | |
| --- | --- | --- | --- | --- |
|  | **DNeasy** | **QIAmp** | **DNeasy** | **QIAmp** |
| Mean | 3.3713 | 3.6288 | 2.3843 | 2.4743 |
| N | 8 | | 7 | |
| Correlation | 0.65 | | 0.85 | |
| Sig. | 0.08 | | 0.015 | |
| t | -2.466 | | -0.906 | |
| df | 7 | | 6 | |
| Sig. (2-tailed) | 0.04 | | 0.4 | |
