# Supplementary material for: Quantification of Cell-Free DNA in Normal and Complicated Pregnancies: Overcoming Biological and Technical Issues
Source: PLoS One. 2014 Jul 2;9(7):e101500. doi: 10.1371/journal.pone.0101500 (PMC4079713; doi:10.1371/journal.pone.0101500)
Supplement: Table S4 — Primers and probes used in the study. (DOCX) [file pone.0101500.s008.docx]

**Supplementary Table S4. Primers and probes used in the study.**

| **Target** | **Primer/probe sequence** | | **Reference** |
| --- | --- | --- | --- |
| *RASSF1A* | F | AGC TGG CAC CCG CTG G | Tong *et al*., 2010 |
|  | R | GTG TGG GGT TGC ACG CG |  |
|  | Probe | VIC-ACC CGG CTG GAG CGT-(MGB) |  |
| *b-Actin* | F | GCA AAG GCG AGG CTC TGT | Hindson *et al*., 2011 |
|  | R | CGT TCC GAA AGT TGC CTT TTA TGG |  |
|  | Probe | FAM-ACC GCC GAG ACC GCG TC-(MGB) |  |
| *RPP30* | F | GAT TTG GAC CTG CGA GCG | Hindson *et al*., 2011 |
|  | R | GCG GCT GTC TCC ACA AGT |  |
|  | Probe | VIC-CTG ACC TGA AGG CTC T-(MGB) |  |
| *SRY* | F | CCA CTT ACC GCC CAT CAA C | Designed in the lab using |
|  | R | AGG TCT TTG TAG CCA ATG TTA CCC | Primer3 Software |
|  | Probe | FAM-ACC GCT ACA(ZEN)GCC ACT GG-(BQH) |  |
