# Supplementary material for: Quantification of Cell-Free DNA in Normal and Complicated Pregnancies: Overcoming Biological and Technical Issues
Source: PLoS One. 2014 Jul 2;9(7):e101500. doi: 10.1371/journal.pone.0101500 (PMC4079713; doi:10.1371/journal.pone.0101500)
Supplement: Table S5 — Normality test (Shapiro-Wilk). (DOCX) [file pone.0101500.s009.docx]

**Supplementary Table S5.** **Normality test (Shapiro-Wilk)**. *p*-values >0.05 indicate a normal distribution.

| **Dataset** | **Statistic** | **df** | **Sig. (*p*)** |
| --- | --- | --- | --- |
| *RPP30*q | .968 | 33 | .416 |
| *RPP30*dd | .971 | 33 | .510 |
| *SRY*q | .950 | 20 | .370 |
| *SRY*dd | .944 | 17 | .363 |
| *RASSF1A*q | .966 | 32 | .395 |
| *RASSF1A*dd | .970 | 26 | .627 |
