# Supplementary material for: Quantification of Cell-Free DNA in Normal and Complicated Pregnancies: Overcoming Biological and Technical Issues
Source: PLoS One. 2014 Jul 2;9(7):e101500. doi: 10.1371/journal.pone.0101500 (PMC4079713; doi:10.1371/journal.pone.0101500)
Supplement: Table S6 — Number of wells revealing specific amplification in qPCR assay in the samples negative for placental DNA according to ddPCR data. (DOCX) [file pone.0101500.s010.docx]

**Supplementary Table S6. Number of wells revealing specific amplification in qPCR assay in the samples negative for placental DNA according to ddPCR data.**

| **Sample** | ***SRY*** | ***RASSF1A*** |
| --- | --- | --- |
| 178 | 2/4 | 1/2 |
| 216 | 4/4 |  |
| 239 |  | 3/4 |
| 243 |  | 2/4 |
| 254 |  | 2/4 |
| 289 |  | 0/4 |
| 290 | 3/4 | 3/4 |
