# Supplementary material for: Quantification of Cell-Free DNA in Normal and Complicated Pregnancies: Overcoming Biological and Technical Issues
Source: PLoS One. 2014 Jul 2;9(7):e101500. doi: 10.1371/journal.pone.0101500 (PMC4079713; doi:10.1371/journal.pone.0101500)
Supplement: Table S7 — Correlation of cfDNA levels with the gestational age at blood draw (GA). (DOCX) [file pone.0101500.s011.docx]

**Supplementary Table S7.** **The association with the gestational age at blood draw (GA).** Cells include R, *p*-value (statistically significant correlations with *p*<0.05 are in bold) and n (number) of samples.

|  | **lg(*RPP30*)q** | **lg(*RPP30*)dd** | **lg(*SRY*)q** | **lg(*SRY*)dd** | **lg(*RASSF1A*)q** | **lg(*RASSF1A*)dd** |
| --- | --- | --- | --- | --- | --- | --- |
| R | **.483** | -.026 | .490 | .083 | .450 | .467 |
| *p*-value | **.006** | .888 | **.039** | .768 | **.013** | **.022** |
| n | **31** | 31 | 18 | 15 | 30 | 24 |
